# Calcium signaling in hepatitis B virus infection and its potential as a therapeutic target

**DOI:** 10.1186/s12964-021-00762-7

**Published:** 2021-08-06

**Authors:** Fanyun Kong, Fulong Zhang, Xiangye Liu, Suping Qin, Xiaoying Yang, Delong Kong, Xiucheng Pan, Hongjuan You, Kuiyang Zheng, Renxian Tang

**Affiliations:** 1 Jiangsu Key Laboratory of Immunity and Metabolism, Department of Pathogenic Biology and Immunology, Xuzhou Medical University, Xuzhou, 221004 Jiangsu China; 2 Imaging Department, The Second Affiliated Hospital of Shandong First Medical University, Taian, Shandong China; 3 Department of Infectious Diseases, The Affiliated Hospital of Xuzhou Medical University, Xuzhou, Jiangsu China; 4 National Demonstration Center for Experimental Basic Medical Sciences Education, Xuzhou Medical University, Xuzhou, Jiangsu China

**Keywords:** Hepatitis B virus, Calcium signaling, Infection, Therapy

## Abstract

As a ubiquitous second messenger, calcium (Ca2+) can interact with numerous cellular proteins to regulate multiple physiological processes and participate in a variety of diseases, including hepatitis B virus (HBV) infection, which is a major cause of hepatitis, fibrosis, cirrhosis, and hepatocellular carcinoma. In recent years, several studies have demonstrated that depends on the distinct Ca2+ channels on the plasma membrane, endoplasmic reticulum, as well as mitochondria, HBV can elevate cytosolic Ca2+ levels. Moreover, within HBV-infected cells, the activation of intracellular Ca2+ signaling contributes to viral replication via multiple molecular mechanisms. Besides, the available evidence indicates that targeting Ca2+ signaling by suitable pharmaceuticals is a potent approach for the treatment of HBV infection. In the present review, we summarized the molecular mechanisms related to the elevation of Ca2+ signaling induced by HBV to modulate viral propagation and the recent advances in Ca2+ signaling as a potential therapeutic target for HBV infection.

**Video Abstract**

**Supplementary Information:**

The online version contains supplementary material available at 10.1186/s12964-021-00762-7.

## Background

The chronic infection of the hepatitis B virus (HBV) is a public health concern and could cause different forms of liver diseases, including hepatitis, fibrosis, cirrhosis, and hepatocellular carcinoma (HCC) around the world [1]. Despite the reduction in carriers of HBV surface antigen due to the use of vaccines, more than billions of people are still suffering from HBV infection. Because HBV is a small DNA virus, the virus heavily depends on host cell factors to facilitate its replication [1, 2]. Calcium (Ca2+) is a ubiquitous second messenger. When Ca2+ flows into cells, it can interact with numerous proteins to control a variety of physiological processes, including survival, proliferation, apoptosis, and autophagy [3, 4]. Besides, the ion was also involved in many different kinds of diseases, such as cancer and viral infection [5, 6]. Especially, the accumulating evidence has highlighted that HBV can modify Ca2+ signaling to create a cellular environment to facilitate its infection [7–9]. Furthermore, the dysregulations of intracellular Ca2+ signaling mediated by the ion channels located in the plasma membrane (PM), endoplasmic reticulum (ER), and mitochondria are mainly responsible for the alteration of Ca2+ homeostasis mediated by the virus [7, 10–12]. Here, we summarize the available data associated with HBV-dependent elevation of intracellular Ca2+ signaling to modulate viral replication and the therapeutic potential of targeting Ca2+ signaling to inhibit HBV infection.

## The role and molecular mechanisms associated with HBV on elevating intracellular Ca2+ levels

The regulation of cytosolic Ca2+ signaling mainly depends on the distinct ion channels located in the PM, ER, as well as mitochondria [3]. For example, relies on voltage-operated Ca2+ channels (VOCCs) and PM store-operated Ca2+ (SOC) channels, the ion enters the cells. Conversely, the PM Ca2+ ATPase (PMCA) channels and Na+ /Ca2+ exchanger (NCX) extrudes the ion from the cytoplasm to the extracellular space. In the cells, Ca2+ sensors and sarco/endoplasmic reticulum ATPase (SERCA) pumps Ca2+ into the lumen of ER, from where Ca2+ is released via inositol 1,4,5-trisphosphate receptor channels (IP3R) and ryanodine receptor (RyR). In addition, mitochondria takes up Ca2+ through voltage-dependent anion channel (VDAC) in the outer mitochondrial membrane (OMM) and the mitochondrial Ca2+ uniporter (MCU) complex in the inner mitochondrial membrane (IMM). Moreover, it extrudes Ca2+ via mitochondrial NCX (mNCX) [4]. If Ca2+ accumulation is overwhelmed in the mitochondrial matrix, the ion is capable of activating the mitochondrial permeability transition pore (mPTP), a non-selective channel across the inner and outer membranes of mitochondria, to transfer Ca2+ and other small molecules to the cytoplasm.

**Fig. 1:**
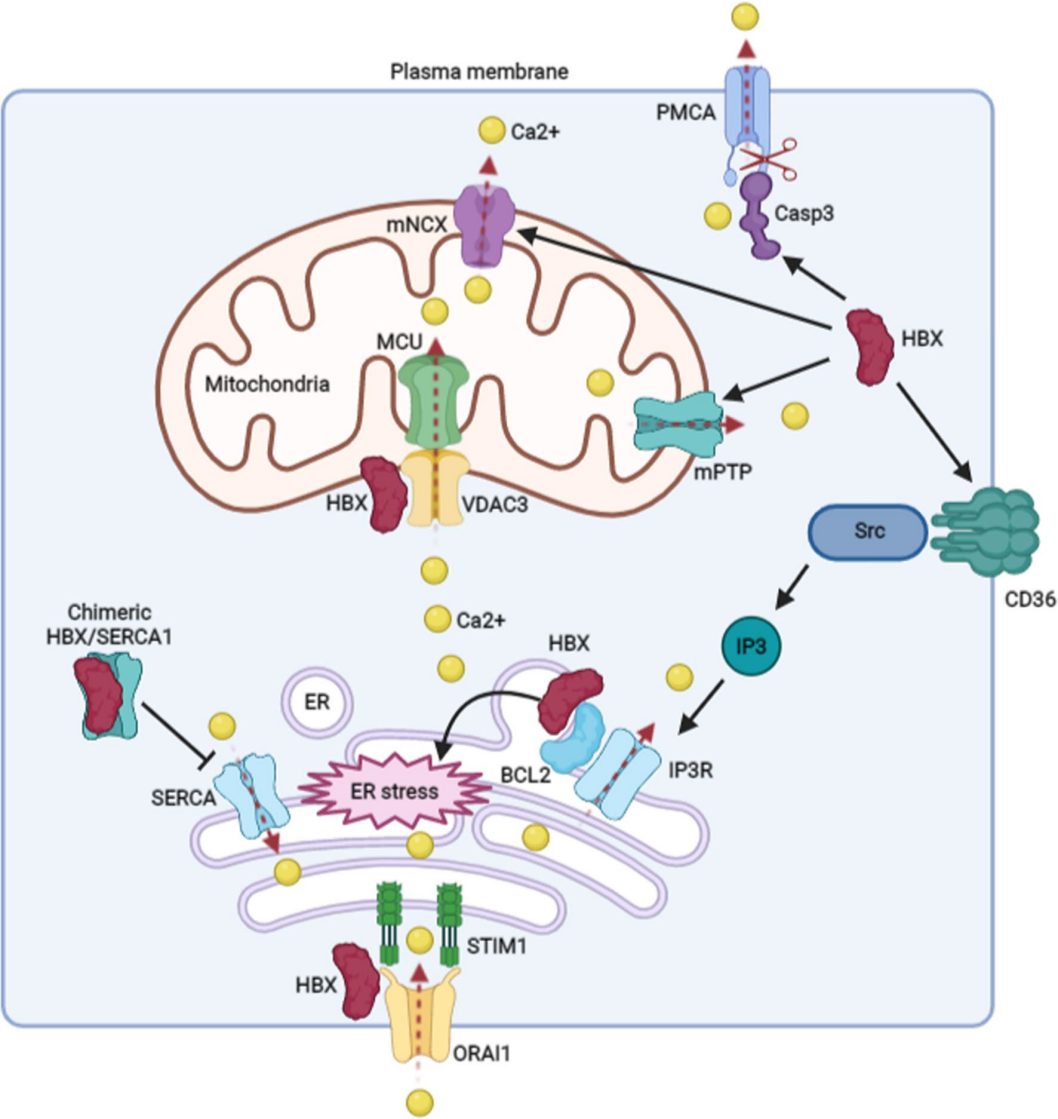
The molecular mechanisms associated with the elevation of Ca2+ signaling mediated by HBX. In detail, HBX promotes the elevation of cellular Ca2+ levels by interacting with ORAI1, which can bind to STIM1 and enhance the flow of Ca2+ into the cytosol. HBX increases the ER stress, which is associated with the release of Ca2+ from the ER. Furthermore, the viral protein enhances the outflow of Ca2+ from the ER through the activation of IP3R by increasing CD36, the molecules can activate Src pathway and induce the production of IP3 to interact with IP3R and then activate IP3R. In addition, the interaction of HBX and Bcl2 maybe also contribute to the activation of IP3R. The chimeric HBX/SERCA1 inhibits the function of SERCA to facilitate the flow of Ca2+ signaling into ER. The viral protein may enhance the move of Ca2+ into mitochondria by interacting with VDAC3, which could bind to MCU to facilitate Ca2+ inflow. HBX also promotes the outflow of Ca2+ from mitochondria by regulating mNCX and mPTP. Besides these, HBX perturbs intracellular Ca2+ outflow to outside of the cells by targeting Casp3, which could cleave PMCA. Ca2+ , calcium; ER: endoplasmic reticulum; PMCA, plasma membrane Ca2+ ATPase; SERCA, sarco/endoplasmic reticulum ATPase; IP3, inositol 1,4,5- triphosphate; IP3R, inositol 1,4,5-trisphosphate receptor; VDAC, voltage-dependent anion channel; MCU, mitochondrial Ca2+ uniporter; mNCX, mitochondrial Na+ /Ca2+ exchanger; mPTP, mitochondrial permeability transition pore; STIM1, stromal interaction molecule protein 1; Casp3: Caspase-3

In hepatocytes, the increase of cytosolic Ca2+ is mainly from the extracellular space, ER, and mitochondria. Among HBV-encoded proteins, HBX is found to be responsible for inducing extracellular Ca2+ influx [11], and the effect of HBX on Ca2+ entry into the cytoplasm from the extracellular space is mainly dependent on SOC channels. The best-characterized SOC channel is the Ca2+ release-activated Ca2+ (CRAC) channel [3]. Recently, ORAI channels, such as ORAI1, -2, and -3, are identified as key molecular components of CRAC channels [4, 5, 13]. Although HBV does not alter the expression levels of SOC channel components, the latest evidence shows that HBX could bind to ORAI1 [13], and this interaction may be contribute to the influx of extracellular Ca2+ into the cells mediated by the virus. Consistent with the expectation, the study from Yang et al. showed that compared to control cells, the levels of cytosolic Ca2+ mediated by HBX in ORAI1-E106A (dominant-negative ORAI1 mutant)-expressing hepatoma cells were inhibited [11]. Besides these, Chami et al. showed that HBX had the capability of inhibiting the activity of PMCA in the PM to avoid the Ca2+ outflux, through activating caspase-3, to cleave PMCA protein [14] (Fig. 1). However, except for ORAI1 and PMCA, whether other kinds of ion channels or transporters situated in the PM are involved in the regulation of cytosolic Ca+ levels mediated by HBV is still unknown.

As the largest intracellular Ca2+ storage, ER plays a very important role in regulating cytosolic Ca2+ levels [15]. Current investigation shows that elevated ER stress is associated with Ca2+ release from the organelle [16]. HBX is observed to situate in the ER and can induce ER stress [17], and the ER stress mediated by HBX maybe contribute to the release of Ca2+ from the ER. Similar to HBX, HBV core mutant, PreS1 and PreS2 mutants also induce ER stress [18, 19], which maybe also facilitate the flow of Ca2+ out of the ER. By using different channel inhibitors, Ca2+ outflow from the ER mediated by HBV was found to be mainly relied on IP3R but not RyR [7]. In addition, the findings from Geng et al. indicate that HBX could bind to Bcl2 to elevate the cytosolic Ca2+ levels [20]. However, the detailed mechanisms remain unidentified. Due to the interaction of Bcl2 with IP3R in ER could block the Ca2+ release mediated by IP3R [21], it is reasonable to speculate that the interaction of HBX with Bcl2 may inhibit the Bcl2-IP3R complex, to activate IP3R and facilitate the release of Ca2+ from ER. Besides these, it has been reported that HBV could integrate into IP3R genes in HCC tissues, whether the integration affects IP3R-mediated ER release of Ca2+ in hepatoma cells still unknown [22]. CD36, a major mediator of cellular free fatty acids uptake, is also required to induce the release of Ca2+ from the ER mediated by HBV [23]. Among the HBV proteins, HBX was considered to be mainly responsible for CD36 overexpression [24]. In addition, CD36 can interact with activated Src and results in inositol 1,4,5- triphosphate (IP3) production. IP3 further binds to IP3R on the ER and may stimulate Ca2+ release to reduce ER Ca2+ storage mediated by HBV, especially by HBX [11, 23] (Fig. 1).

Within the cells, reduced ER Ca2+ storage could activate Ca2+ sensors or SECAR channels to uptake Ca2+ [3, 25]. On the one hand, the Ca2+ sensor stromal interaction molecule protein 1 (STIM1) situated in the ER could induce the activation of the SOC channels through binding to ORAIl in the PM, and the interaction of STIM1 and ORAIl facilitates the transfer of Ca2+ from extracellular space into cytosol, and this process is known as store-operated calcium entry (SOCE) [25]. It has been proposed that Ca2+ influx mediated by HBX may be dependent on the binding of HBX with STIM1-ORAIl complexes [13]. In addition to HBX, HBV PreS2-mutant large surface antigen is also capable of recruiting STIM1-resident ER toward ORAIl in PM to promote the interaction of STIM1 with ORAI1 and induce SOCE [26]. On the other hand, the reduced ER Ca2+ storage activates SERCA, which transfers Ca2+ from the cytoplasm into ER [3]. Although it has been reported to the activation of SERCA is associated with HBV replication [7], the study from Chami et al. showed that HBX can integrate into the SERCA1 gene, and forms chimeric HBX/SERCA1 transcripts in HCC tissues. In vitro analysis showed that HBX/SERCA1 proteins localized to ER, and block the function of SERCA1 to induce ER Ca2+ depletion in hepatoma cells [27].

Mitochondria is a vital cellular organelle with the function of participating in the regulation of cellular Ca2+ signaling to promote the tricarboxylic acid cycle and ATP synthesis [28]. The growing evidence shows that HBX contributes to mitochondrial Ca2+ uptake [11]. Ca2+ fluxing into mitochondria is mainly dependent on VDAC in OMM, and MCU with interactions of several regulatory subunits, including MICU1, MICU2, and EMRE in IMM [3, 4]. It has been observed that HBV is capable of increasing MICU1 expression, and HBX could interact with VDAC3 [12, 29] (Fig. 1). The regulation of MICU1 and VDAC3 mediated by the virus maybe facilitate mitochondrial Ca2+ uptake. Importantly, the findings from Casciano et al. indicates that HBV stimulates mitochondrial Ca2+ influx during the ion releases from the ER and/or Ca2+ entry through the SOC channels in the PM. The mitochondrial Ca2+ uptake is capable of dampening Ca2+ -mediated inhibition of further Ca2+ release from ER and/or Ca2+ entry through SOC channel, thereby prolonging Ca2+ entry into the cytoplasm to increase the levels of cytosolic Ca2+ [12]. Once Ca2+ is overloaded in mitochondria, mNCX and mPTP could be triggered to open and transfer Ca2+ into the cytoplasm [28]. Furthermore, it has been proposed that both mNCX and mPTP are involved in Ca2+ alteration mediated by HBX in mitochondria [8, 30]. Together, these evidence suggests that HBV, especially HBX, influences the activity of distinct Ca2+ channels in the PM, ER, and mitochondria to facilitate the regulation of cytosolic Ca2+ levels. An in-depth understanding of the details associated with the regulation of Ca2+ channels induced by the virus could help us develop suitable inhibitors to restrict these target channels and facilitate the treatment of HBV infection.

## The role and molecular mechanisms associated with Ca2+ signaling on HBV life cycle

As a partially double-stranded DNA virus, HBV contains 4 overlapping open reading frames (ORFs): P, S, C, and X. The P ORF encodes Pol protein. S ORF encodes S, preS1, and preS2 domains to construct the LS (large surface), MS (middle surface), and S (small surface) proteins. Two genes locate in C ORF and are responsible for the expression of HBc and precore proteins. The X region is the least ORF, and it is capable of encoding HBX protein. After the virus interacts with the receptor sodium taurocholate cotransporting polypeptide (NTCP), it enters into the cells and uncoated. Sequentially, the genome of the virus was transferred into the nucleus and converted into covalently closed circular DNA (cccDNA). Next, the cccDNA forms a minichromosome and transcripts to 4 lengths of viral mRNA, including 3.5 kb preC mRNA and pregenomic RNA (pgRNA), 2.4 and 2.1 kb envelope mRNAs, and 0.7 kb X mRNA. The preC mRNA encodes precore protein, which is further cleaved and secrets as HBe. pgRNA translates HBc and Pol proteins as well as acts as a template for viral replication. The 2.4 and 2.1 kb envelope mRNAs encode LS, MS, and S proteins. Additionally, the X mRNA translates HBX protein. After these viral RNAs translate into HBV proteins, the pgRNA is encapsulated into core particles, and further reversely transcribes into viral DNA. Then, the viral particles containing HBV DNA are enveloped and secret from the cells [31, 32].

**Fig. 2:**
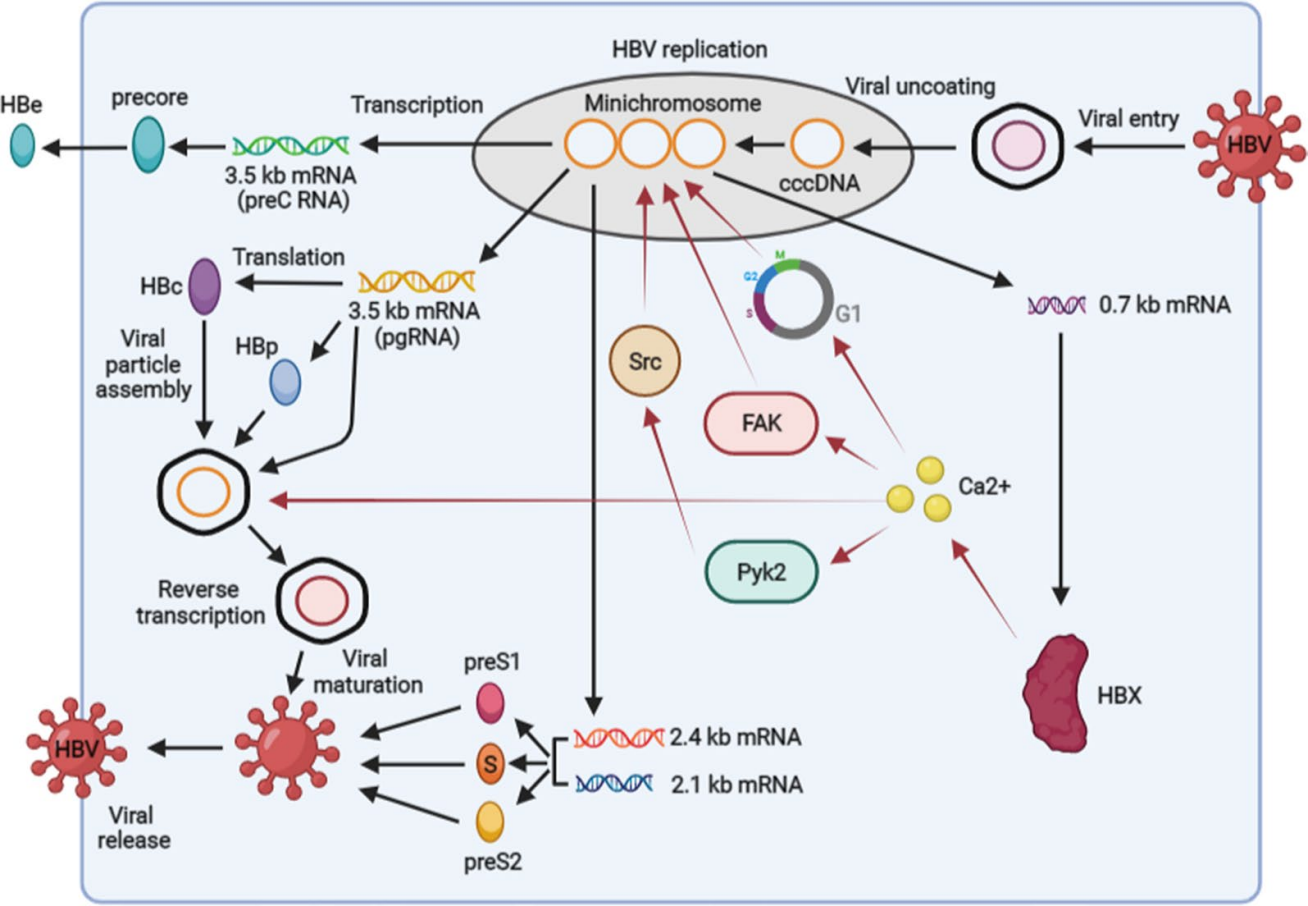
The molecular mechanisms associated with Ca2+ signaling mediated by HBX to regulate HBV life cycle. HBX elevates the levels of Ca2+ to activate Pyk2/Src and FAK pathways, and regulate the cell cycle to stall in G1 phase to promote HBV replication. In addition, the Ca2+ signaling mediated by HBX facilitates viral core assembly. cccDNA, covalently closed circular DNA; pgRNA, pregenomic RNA

In the past years, several works have been focused on understanding the role of Ca2+ signaling mediated by HBV in accelerating its replication. As a virus non-structural protein, HBX plays a critical role in HBV replication [31, 32]. A recent study reports that, via Ca2+ signaling, fatty acids could promote the stabilization of HBX [33], and this role mediated by fatty acids maybe contribute to the HBV replication. More importantly, several studies have demonstrated that the Ca2+ -dependent signaling mediated by HBX contributes to HBV replication (Fig. 2). Mechanistically, it has been proposed that Pyk2/Src and FAK signals were involved in the replication of HBV [8, 34]. Inhibition of cytosolic Ca2+ blocks HBX-dependent activation of Pyk2/Src and FAK signals, and in turn, inhibits HBV replication. In addition to distinct signals, HBX also could use Ca2+ signaling to regulate the cell cycle to stall in G1 to stimulate virus replication [35, 36]. Especially, the alteration of Ca2+ levels mediated by SOCE, ER, and mitochondria is observed to be involved in the replication of HBV mediated by HBX, because knocking down ORAI1 to block SOCE, inhibiting IP3R situated in ER, or suppressing mPTP located in mitochondria, could restrict HBX-dependent viral replication [11, 35].

The innate immune response is the first line of host defense against HBV infection. Binding to the interferon-α/β receptor, the innate immune molecule interferon (IFN) could initiate Janus kinase/signal transducer and activator of transcription (JAK/STAT) pathways to induce the expression of IFN-stimulated genes (ISGs) to further eliminate the virus [32]. However, HBV has the capability of resisting IFN-mediated innate immunity to facilitate its replication through increasing the expression of intracellular Ca2+ signaling-modulated proteins, including Calreticulin, an ER luminal protein whose main functions are based on the binding with Ca2+ in hepatocytes [37]. Detailed mechanisms suggest that Calreticulin mediated by HBV could inhibit IFN-α production by reducing the nuclear translocation of IFN regulatory factor-7. Moreover, Calreticulin suppresses the antiviral activity of IFN-α is related to the suppression of STAT1 activation and the decrease in the expression of two ISGs, protein kinase R and 2',5′-oligoadenylate synthetase.

To date, the current studies also demonstrated that increased Ca2+ mediated by HBX enhanced HBV core assembly (also known as capsid assembly) in hepatocytes [38] (Fig. 2). However, the molecular mechanisms related to HBX-dependent core assembly induced by Ca2+ signaling were not well explored so far. As a viral polymerase, HBV Pol protein has an important role in viral DNA replication and reverse transcription in the HBV life cycle [31, 32]. Choi et al. showed that HBV Pol interacted with S100A10, a Ca2+ -modulated protein, and the interaction inhibit the activity of HBV Pol and transport HBV Pol to PML nuclear bodies (PML NBs) to inhibit viral replication. In the aspect of mechanisms, the role of S100A10 on the transport of HBV Pol to PML NBs could be regulated by intracellular Ca2+ . Elevated cytosolic Ca2+ is capable of blocking the effect of S100A10 on the transport of Pol protein to PML NBs [39]. Together, the evidence presented here suggests that the cellular factors controlled by Ca2+ signaling participate in different steps of the viral life cycle during HBV replication. Therefore, further explore the effect of Ca2+ signaling and associated molecules on other steps of HBV life may deepen the understanding of Ca2+ signaling-dependent mechanisms related to viral replication.

## Ca2+ signaling as a potential therapeutic target in HBV infection

**Fig. 3:**
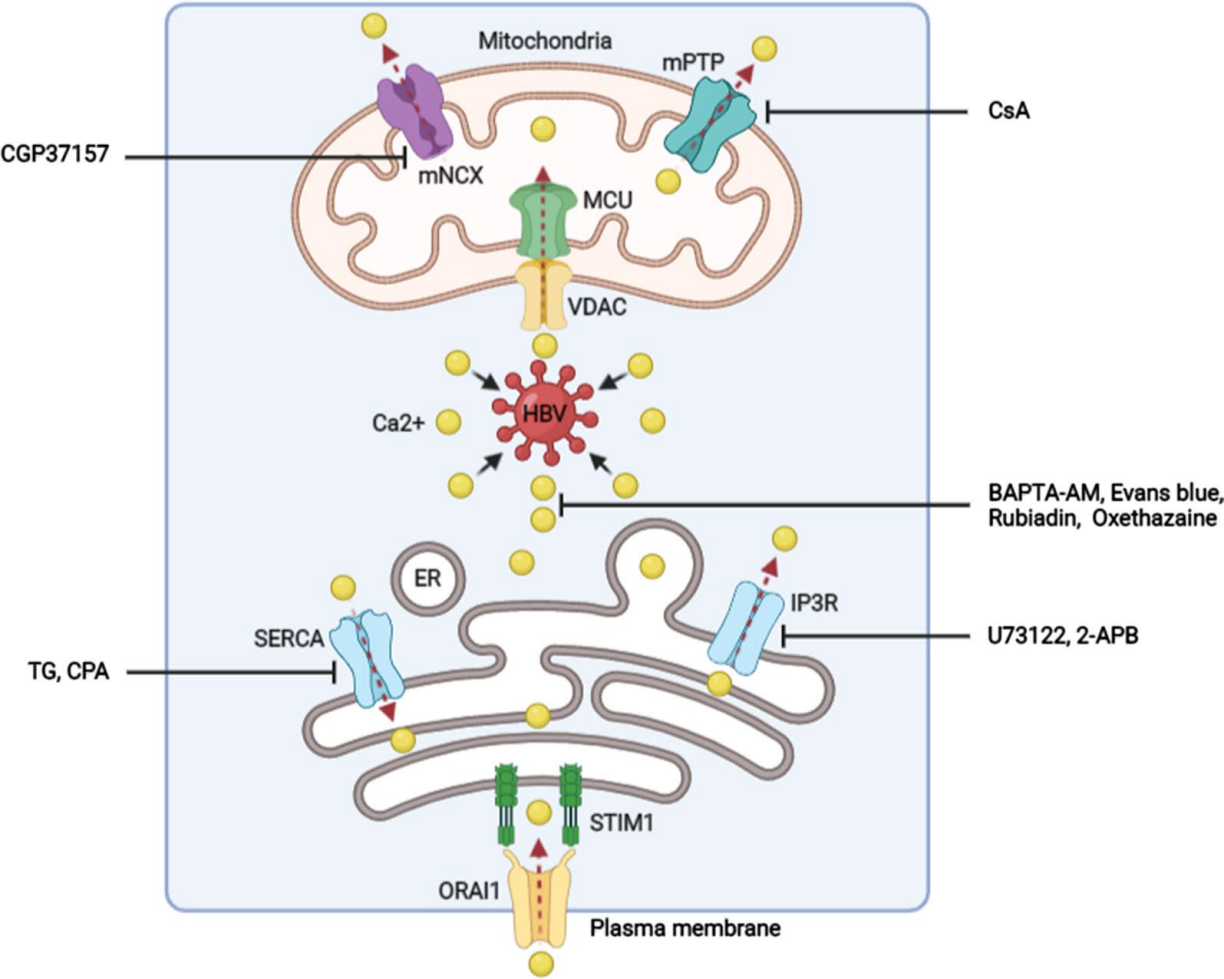
The pharmaceuticals inhibit HBV infection by targeting Ca2+ signaling. CsA, cyclosporine A; TG, thapsigargin; CPA, cyclopiazonic acid

Because of the importance of Ca2+ signaling in HBV infection, the increasing evidence has demonstrated that controlling intracellular Ca2+ or Ca2+ channels by suitable pharmaceuticals is a potential strategy to control HBV replication (Fig. 3). For instance, chelation of cytosolic Ca2+ with BAPTA-AM [8, 35], and Rubiadin, a regent isolated from prismatomeris connate [40], has a significant antagonistic effect on viral replication. Recently, the emerging evidence indicates that the inhibition of mitochondria channels, including targeting mNCX channels with CGP37157 [8] or suppressing mPTP with cyclosporine A (CsA) can block HBX-dependent HBV replication [41]. Besides these, treating SERCA with thapsigargin (TG) and cyclopiazonic acid (CPA), or targeting IP3R with U73122, or 2-aminoethoxydiphenyl (2-APB) to restrict the function of Ca2+ channels in ER [7, 12], also have a significant inhibitory effect in HBV replication. Moreover, the inhibition of HBV replication mediated by different pharmaceuticals to target Ca2+ signaling was associated with the inactivation of Pyk2/Src and FAK signals mediated by HBX [8, 34].

As mentioned, HBV core assembly is promoted by Ca2+ signaling [38]. The work from Choi et al. demonstrates that the inhibition of cytosolic Ca2+ levels by BAPTA-AM, or block mPTP open in mitochondria by CsA could reduce the assembly of viral core protein in viral-transfected hepatoma cells [38]. Similarly, Xiao et al. show that Evans Blue, an FDA-approved agent used for treating blood–brain barrier disruption, suppresses HBV core assembly via targeting the cytosolic Ca2+ signaling [42]. Interestingly, both CsA and Evans Blue are also involved in the inhibition of HBV entry into host cells by controlling the binding of viral envelope protein to the membrane transporter NTCP [42, 43]. Another FDA-approved agent, oxethazaine, is shown to inhibit HBV replication by blocking the self-assembly of HBV core protein as well. Moreover, oxethazaine has the capability of inhibiting the replication of lamivudine/entecavir-dual-resistant and adefovir-resistant HBV mutants. Besides, the reduction of cytosolic Ca2+ concentration and Pyk2 activation by oxethazaine [44], is responsible for its inhibition on HBV core assembly.

## Conclusions

The evidence presenting here indicates that HBV can utilize multiple mechanisms to elevate the levels of intracellular Ca2+ . In turn, the increased cytosolic Ca2+ is capable of facilitating viral replication in several manners. Moreover, the available evidence suggests that the use of different kinds of pharmaceuticals that targeting Ca2+ signaling is a potent strategy for HBV. Until now, significant progress has been made in identifying the potential targets, including Ca2+ channels and Ca2+ dependent proteins, for pharmacological intervention [5]. An in-depth understanding of the activation of Ca2+ signaling induced by HBV can develop novel therapeutic approaches to control the virus.

Specifically, HBX has been demonstrated to participate in viral replication [31, 32], although the exact mechanisms remain not fully elaborated. Our reviewed studies support that the elevation of Ca2+ signaling and related functions mediated by HBV is mainly dependent on HBX. On the one hand, through regulating the activation and expression of multiple Ca2+ channels, including ORAI1, IP3R, and mPTP in the PM, ER, and mitochondria (Fig. 1), the viral protein could elevate the levels of Ca2+ in the cytoplasm. On the other hand, HBX contributes to viral replication via Ca2+ signaling-dependent activation of Pyk2/Src and FAK pathways. Based on Ca2+ signaling, HBX also facilitates core assembly (Fig. 2), despite the detailed mechanisms are not defined. To better understand the role of Ca2+ signaling in the HBV life cycle mediated by HBX, more investigations on the complicated interactions between HBX and Ca2+ signaling are deserved.

Besides these, our knowledge on the role of Ca2+ signaling mediated by HBX on viral replication was mainly from the in vitro cellular models. The effect of Ca2+ signaling on HBX-dependent HBV replication in animal models as well as patients with HBV infection is unknown. Furthermore, although Ca2+ signaling facilitates HBV replication, the influence of cytosolic Ca2+ signaling on the development of HBV-associated diseases, including hepatitis, cirrhosis, and HCC, is not well clarified. Therefore, future studies are needed to provide more insights into the biological processes associated with the alteration of Ca2+ signaling mediated by HBV.

## Data Availability

Not applicable.

## Abbreviations

Ca2+: Calcium
HBV: Hepatitis B virus
ER: Endoplasmic reticulum
IFN: Interferon
ORFs: Open reading frames
cccDNA: Covalently closed circular DNA
pgRNA: Pregenomic RNA
PM: Plasma membrane
PMCA: PM Ca2+ ATPase
VOCCs: Voltage-operated Ca2+ channels
NCX: Na+/Ca2+ exchanger
SERCA: Sarco/endoplasmic reticulum ATPase
IP3R: Inositol 1,4,5-trisphosphate receptor channels
VDAC: Voltage-dependent anion channel
OMM: Outer mitochondrial membrane
MCU: Mitochondrial Ca2+ uniporter
IMM: Inner mitochondrial membrane
mNCX: Mitochondrial NCX
mPTP: Mitochondrial permeability transition pore
CRAC: Ca2+ release-activated Ca2+
IP3: Inositol 1,4,5-triphosphate
STIM1: Transmembrane stromal interaction molecule protein 1
CsA: Cyclosporine A
TG: Thapsigargin
CPA: Cyclopiazonic acid
